# One-Step Synthesis of *N*-Succinimidyl-4-[^18^F]Fluorobenzoate ([^18^F]SFB)

**DOI:** 10.3390/molecules24193436

**Published:** 2019-09-22

**Authors:** Ida Nymann Petersen, Jacob Madsen, Christian Bernard Matthijs Poulie, Andreas Kjær, Matthias Manfred Herth

**Affiliations:** 1 Department of Clinical Physiology, Nuclear Medicine & PET, University Hospital (Rigshospitalet), Blegdamsvej 9, DK-2100 Copenhagen, Denmark; ida.nymann.petersen@regionh.dk; 2 Cluster for Molecular Imaging, Department of Biomedical Sciences, University of Copenhagen, Blegdamsvej 9, DK-2100 Copenhagen, Denmark; Jacob.Madsen@regionh.dk; 3 Department of Drug Design and Pharmacology, Faculty of Health and Medical Sciences, University of Copenhagen, Jagtvej 160, DK-2100 Copenhagen, Denmark; christian.poulie@sund.ku.dk

**Keywords:** *N*-succinimidyl-4-[^18^F]fluorobenzoate, [^18^F]SFB, iodonium ylides, fluorine-18, PET

## Abstract

Herein, we present a one-step labeling procedure of *N*-succinimidyl-4-[^18^F]-fluorobenzoate ([^18^F]SFB) starting from spirocyclic iodonium ylide precursors. Precursor syntheses succeeded via a simple one-pot, two-step synthesis sequence, in yields of approximately 25%. Subsequent ^18^F-nucleophilic aromatic labeling was performed, and radiochemical incorporations (RCCs) from 5–35% were observed. Purification could be carried out using HPLC and subsequent solid phase extraction. Radiochemical purity (RCP) of >95% was determined. The total synthesis time, including purification and formulation, was no longer than 60 min. In comparison to the established 3-step synthesis route of [^18^F]SFB, this one-step approach avoids formation of volatile radioactive side-products and simplifies automatization.

## 1. Introduction

**Figure 1:**
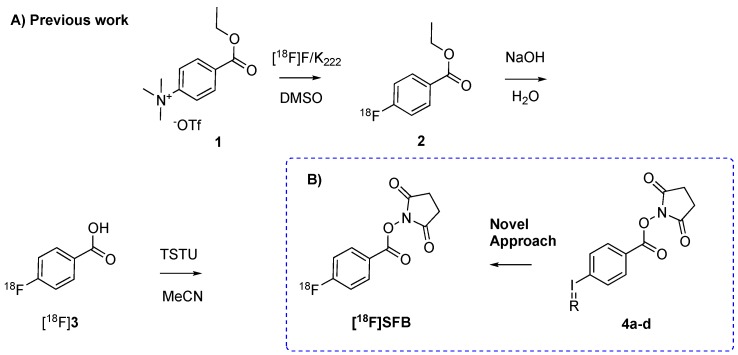
Standard procedure vs. novel one-step approach; (**A**) 3-step synthesis of *N*-Succinimidyl-4-[^18^F]Fluorobenzoate ([^18^F]SFB), (**B**) Novel synthesis approaches based on spirocyclic iodonium ylide precursors.

*N*-succinimidyl-4-[^18^F]fluorobenzoate ([^18^F]SFB) is one of the most used prosthetic groups for labeling of proteins and peptides. High conjugation yields and metabolic stability of the formed construct facilitate its widespread use [1,2,3,4,5,6,7]. For example, [^18^F]SFB has been successfully applied in the synthesis of inhibited factor VII [1], RGDs [4], or serum albumin [6]. Current synthesis approaches are routinely based on a 3-step synthesis sequence starting with an ^18^F-aromatic nucleophilic substitution (S_N_Ar) of 4-(ethoxycarbonylphenyl)trimethylammonium triflate (1), followed by a hydrolysis step of the corresponding ethyl ester (2) to the benzoic acid derivative [^18^F]3 ([^18^F]FBA) and finalized by formation of [^18^F]SFB (Figure 1) [1,2,3,4,5,6,7]. In general, automation of 3-step synthesis procedures can be challenging, and not all available synthesis modules support 3-step synthesis procedures. As such, the synthesis of [^18^F]SFB cannot easily be implemented at all production sites. The 3-step synthesis of [^18^F]SFB is further complicated since relatively high amounts of a volatile radioactive side-product, probably [^18^F]FMe, are released [8]. This released side-product can limit the number of possible syntheses repetitions before permitted release levels are reached. In this respect, one-step, direct labeling conditions to synthesize [^18^F]SFB are highly desirable. Unfortunately, standard conditions, which use relatively high amounts of base, result in hydrolysis of the active ester. As such, solely [^18^F]3 is formed. Several attempts have been carried out to circumvent the aforementioned 3-step procedure. This includes labeling of a (thiophen-2-yl)iodonium trifluoroacetate salt precursor [9], a TMP-iodonium-precursor [10], or boronic pinacol ester precursor [11]. None of the described methods report an isolation step of the final product. Moreover, reports make use of aliquot labeling conditions that can usually not easily be translated to one-step procedures with similar radiochemical incorporations (RCCs) or radiochemical yields (RCYs) [12].

Here, we report our efforts towards a one-step radiolabeling procedure to synthesize [^18^F]SFB. We envisaged that spirocyclic iodonium ylide precursors bear the possibility to reach this goal. Spirocyclic iodonium ylides are reported to result in high RCCs and in good RCYs. Furthermore, only short reaction timeframes are necessary [12,13,14]. As such, we hypothesized that the nucleophilic attack of the [^18^F]fluoride anion on the ylide leaving group could be faster than the competing hydrolysis of the active ester. Subsequent quenching of the hydrolysis process shortly after the nucleophilic attack would lead to the desired product, [^18^F]SFB. Moreover, the proposed strategy should prevent the formation of volatile side-products.

## 2. Results and Discussion

**Figure 2:**
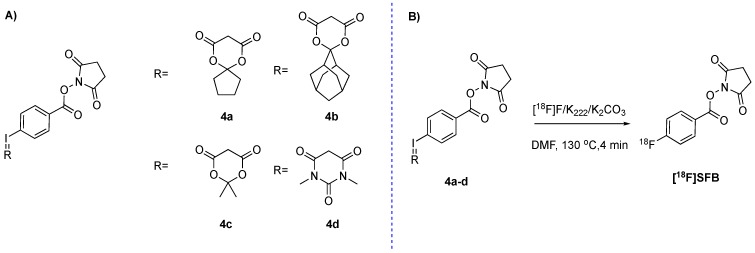
(**A**) Four different precursors were synthesized to find the best precursor for one-step formation of [^18^F]SFB. (**B**) General labeling scheme.

In order to develop a one-pot, ^18^F-direct labeling strategy of [^18^F]SFB, four spirocyclic iodonium ylide precursors 4a–d were synthesized according to literature procedures (Figure 2A) [15]. Precursors 4a–d were obtained in an overall chemical yield of approximately 25%. Aliquot labeling experiments were carried out to investigate the capability of 4a–d to incorporate fluoride-18. As a starting point, we applied ^18^F-labeling conditions that recently have been shown to result in high RCCs within short reaction times (Figure 2B, Supplementary Table S1) [12]. Quenching of the hydrolysis process was conducted by adding 1.5% AcOH. During these experiments, the optimal time point in respect of maximum fluorine-18 incorporation and minimizing [^18^F]3 formation was determined. An optimum was reached after 5 min as longer reaction times lead to an increase in [^18^F]3 formation (Supplementary Table S1). Precursor 4a and 4c resulted in a maximum RCC of approximately 35%, whereas 4b and 4d only resulted in RCCs around 5% (Table 1). These initial results encouraged us to investigate the reliability of the reaction. Precursor 4a was selected as a model compound for this purpose. A high variability was detected that spanned from 1–33% RCC with a mean of 17.8 ± 17% (*n* = 26). We believe that this variability is due to different basicity values stemming from slightly different water content and base concentration within the final reaction mixture. Absolute water content and base concentration are very difficult to control within standard labeling procedures and can vary. In turns, sensitive reactions can be strongly influenced by these concentration differences. In our case, we believe that these differences could lead to an accelerated hydrolysis step and, as such, higher amounts of [^18^F]3 are formed. To minimize this, we investigated the influence of various bases, such as tetraethylammonium bicarbonate (TEAB), KHCO_3_, or Cs_2_CO_3_. Only trace amounts of [^18^F]SFB could be detected using these bases (Supplementary Table S2). The addition of (2,2,6,6-tetramethylpiperidin-1-yl)oxyl (TEMPO), which is reported to increase the RCC of this type of reactions [12], and the change of phase transfer catalyst did not improve the yield, either. Furthermore, the use of *N,N*-dimethylformamide (DMF) turned out to be essential for the reaction (Supplementary Table S2). In an attempt to reduce the water content within the final reaction mixture, as well as to accelerate ^18^F-incorporation, microwave heating was applied; unfortunately, it was unsuccessful. Consequently, we did not succeed in improving the variability of the reaction.

**Figure 3:**
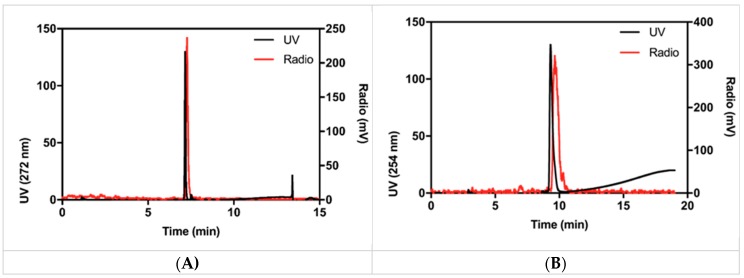
(**A**) [^18^F]SFB synthesized in 1-step after HPLC purification. Reference compound is co-injected for chemical identification purposes. (**B**) [^18^F]SFB reacted with FVIIai and purified with a PD10 column.

Despite these failed attempts, we were interested in upscaling the reaction. Precursors 4a and 4c were selected for this endeavor. A non-decay corrected RCY of 1–15% at the end of synthesis with a low molar activity of 2 GBq/μmol and a radiochemical purity (RCP) of >95% could be reached (Figure 3A). The total reaction time was below 60 min, including HPLC purification and final formulation. No release of any radioactive side-product was observed. Figure 3 shows a typical HPLC chromatogram of [^18^F]SFB after purification and formulation. The displayed sample was spiked with reference compound for identification purposes. Finally, we tested if [^18^F]SFB can indeed be used for labeling purposes. A 50 kDa protein (Active site-inhibited FVII (FVIIai) was successfully labeled with [^18^F]SFB. The product, [^18^F]FVIIai, was isolated in a satisfying RCY of 11% compared to the starting activity amount of [^18^F]SFB (Figure 3B).

In comparison to commonly used 3-step procedures, the new method resulted in relatively low, but for preclinical application, acceptable non-decay corrected RCY (2–15%). RCYs showed a high variability. Average RCY was 4%, lowest 2%. Comparable RCP and synthesis time were achieved with the new method. Table 2 compares the results of this work towards published 3-step labeling procedures. Finally, labeling of FVIIai resulted in slightly lower RCY compared to the use of [^18^F]SFB produced in a 3-step labeling approach (11% vs. 25 ± 9%) [1].

## 3. Materials and Methods

Solvents and reagents were purchased from Sigma Aldrich (Darmstadt, Germany) or Thermo Fisher Scientific (Waltham, MA, USA). Reference compound SFB was purchased from ABX advanced biochemical compounds (Radberg, Germany). 400 MHz ^1^H-NMR and diffusion-ordered spectra were recorded on a Bruker Avance III HD 400 spectrometer (Billerica, MA, USA) at room temperature. Chemical shifts are reported in ppm and were referenced to the solvent residual signal. The analysis of the ^1^H-NMR spectra was performed using the software MestReNova v12.0.0 (Mestrelab Research S.L., Santiago de Compostela, Spain)). Thin-layer chromatography (TLC) was carried out using normal phase plates (silica gel 60 coated with fluorescent indicator F254s) from Merck (Darmstadt, Germany). The fraction of radioactivity on the TLC-plates was measured with an instant imager from Packard InstantImager (Detroit, MI, USA) and analyzed by Optiquant software v1.2 (PerkinElmer, Waltham, MA, USA). Analytical HPLC was performed on a Dionex (Sunnyvale, CA, USA) system connected to a P680A pump, a UVD 170U detector, and a radiodetector from Scansys Laboratorieteknik (Vanløse, Denmark). HPLC control and spectra processing were done with Chromeleon 6.8 software (Waltham, MA, USA).

### 3.1. General Procedure to Formation of Spirocyclic SFB Precursors

2,5-dioxopyrrolidin-1-yl 4-iodobenzoate (120 mg, 0.35 mmol) was dissolved in acetonitrile (ACN) (0.2 mL) and added to a solution of Selectfluor (140 mg, 1.5 eq.) and Me_3_SiOAc (0.21 mL, 2.7 eq.) in ACN (1.5 mL). The vial with the solution was capped and stirred for 36 h at 75 °C. This mixture was then cooled to RT. The desired acid (1 eq) was dissolved in a solution of 10% aq. Na_2_CO_3_ (0.2 mL) and ACN (0.1 mL), added to the former mixture, and stirred for 1 h. Afterwards, 5 mL water was added, and the resulting mixtures extracted with 3 × DCM (15 mL). The product was purified via flash column chromatography, as specified.

### 3.2. 2,5-dioxopyrrolidin-1-yl 4-((7,9-dioxo-6,10-dioxaspiro[4.5]decan-8-ylidene)- λ3-iodaneyl)benzoate *(**4a**)*

Produced via the general procedure, using 6,10-dioxaspiro[4.5]decane-7,9-dione (60 mg, 1 eq) as the corresponding acid, the product was purified via flash column chromatography starting from 100% n-Hep to 50% DCM in n-Hep, followed by 10:45:45 EtOH:n-Hep:DCM to give 2,5-dioxopyrrolidin-1-yl 4-((7,9-dioxo-6,10-dioxaspiro[4.5]decan-8-ylidene)-λ3-iodaneyl)benzoate (4a) 45 mg (25%). R_f_ 0.65 (10% MeOH in DCM). The compound was stored at −18 °C. ^1^H-NMR (400 MHz, DMSO): δ 8.14 (dd, 2H) 8.06 (dd, 2H), 2.90 (s, 4H), 2.01 (m, 4H), 1.70 (m, 4H); ^13^C-NMR (101 MHz, DMSO): δ 170.1, 163.5, 161.1, 133.1, 132.0, 126.3, 123.7, 112.3, 58.9, 36.8, 25.5, 22.8.

### 3.3. 2,5-dioxopyrrolidin-1-yl 4-(((5r,7r)-4′,6′-dioxospiro[adamantane-2,2′-[1,3]dioxan]-5′-ylidene)- λ3-iodaneyl)benzoate *(**4b**)*

Produced via the general procedure, using (5r,7r)-spiro[adamantane-2,2′-[1,3]dioxane]-4′,6′-dione (138 mg, 1 eq) as the corresponding acid, the product was purified via flash column chromatography starting from 100% n-Hep to 50% DCM in n-Hep, followed by 10:45:45 EtOH:n-Hep:DCM to give 2,5-dioxopyrrolidin-1-yl 4-(((5r,7r)-4′,6′-dioxospiro[adamantane-2,2′-[1,3]dioxan]-5′-ylidene)-λ3-iodaneyl)benzoate (4b) 25 mg (7%). R_f_ 0.44 (10% MeOH in DCM). The compound was stored at −18 °C. ^1^H-NMR (400 MHz, CDCl_3_): δ 8.16 (dd, 2H) 8.02 (dd, 2H), 2.90 (s, 4H), 2.44 (s, 2H), 2.17 (m, 4H)1.87 (s, 2H), 1.73 (m, 5H); ^13^C-NMR (101 MHz, DMSO): δ 170.6, 163.0, 161.6, 139.1, 138.1, 133.5, 132.5, 126.8, 124.1, 105.8, 58.2, 37.0, 35.3, 33.7, 26.4, 26.0.

### 3.4. 2,5-dioxopyrrolidin-1-yl 4-((2,2-dimethyl-4,6-dioxo-1,3-dioxan-5-ylidene)-λ3-iodaneyl)benzoate *(**4c**)*

Produced via the general procedure, using 2,2-dimethyl-1,3-dioxane-4,6-dione (41 mg, 1 eq) as the corresponding acid, the product was purified via flash column chromatography starting from 100% n-Hep to 50% DCM in n-Hep, followed by 10:45:45 EtOH:n-Hep:DCM to give 2,5-dioxopyrrolidin-1-yl 4-((2,2-dimethyl-4,6-dioxo-1,3-dioxan-5-ylidene)- λ3-iodaneyl)benzoate (4c) 85 mg (62%). R_f_ 0.51 (10% MeOH in DCM). The compound was stored at −18 °C. ^1^H-NMR (400 MHz, DMSO): δ 8.14 (dd, 2H) 8.04 (dd, 2H), 2.90 (s, 4H), 1.59 (s, 6H); ^13^C-NMR (101 MHz, DMSO): δ 170.1, 162.9, 161.1, 133.3, 132.0, 126.3, 123.8, 102.9, 58.2, 25.6, 25.5. 

### 3.5. 2,5-dioxopyrrolidin-1-yl 4-((1,3-dimethyl-2,4,6-trioxotetrahydropyrimidin-5(2H)-ylidene)- λ3-iodaneyl)benzoate *(**4d**)*

Produced via the general procedure, using 1,3-dimethylpyrimidine-2,4,6(1H,3H,5H)-trione (45 mg, 1 eq) as the corresponding acid, the product was purified via flash column chromatography starting from 100% n-Hep to 50% DCM in n-Hep, followed by 10:45:45 EtOH:n-Hep:DCM to give 2,5-dioxopyrrolidin-1-yl 4-((1,3-dimethyl-2,4,6-trioxotetrahydropyrimidin-5(2H)-ylidene)- λ3-iodaneyl)benzoate (4d) 50 mg (35%). R_f_ 0.50 (10% MeOH in DCM). The compound was stored at −18 °C. ^1^H-NMR (400 MHz, DMSO): δ 8.10 (dd, 2H) 8.06 (dd, 2H), 3.18 (s, 6H), 2.89 (s, 4H); ^13^C-NMR (101 MHz, DMSO): δ 170.1, 161.1, 160.9, 152.6, 133.0, 132.0, 126.2, 123.5, 70.1, 28.5, 25.5.

### 3.6. Labeling of [^18^F]SFB – Automatization

[^18^F]Fluoride was produced via a (p,n)-reaction on a CTI Siemens cyclotron by irradiating [^18^O]H_2_O with 11 MeV protons. An anion exchange resin (Sep-Pak Light Waters Accell Plus QMA cartridge (Milford, MA, USA)) was washed with EtOH (20 mL), 0.5 M K_2_CO_3_ (aq) (10 mL) and water (20 mL) and dried with air. Then, the aqueous [^18^F]fluoride solution was passed through this exchange resin and the resin eluted with a Kryptofix_222_ (K222: 4,7,13,16,21,24-hexaoxa-1,10diazabicyclo[8.8.8]hexa-cosane) 10 mg, 1.2 mg K_2_CO_3_ in 0.1 mL H_2_O, and 0.6 mL MeOH). The resulting mixture was then gently concentrated to dryness at 90 °C by azeotropic drying with 2 × ACN (0.6 mL), under a nitrogen stream for 20 min, to give no-carrier-added K[^18^F]F–K_222_ complex as a white semi-solid residue.

Precursor 4a (5 mg) was dissolved in 0.3 mL DMF. This mixture was added to the dried [^18^F]FK/K_222_ complex and heated to 135 °C for 4 min. Then this mixture was transferred to 2 mL of 1.5% AcOH (aq.) and, afterwards, purified via semi-preparative HPLC (LUNA (phenomenex) 250 × 10 mm, 5 µm, 50:50 ACN:H_2_O (0.1% TFA) 3 mL/min, RT: 700 sec [^18^F]SFB). The fraction containing [^18^F]SFB was collected into a vial containing 30 mL water. The product was first trapped on the SepPak plus cartridge and, afterwards, eluted with ACN (2 mL) and, finally, evaporated to dryness under a stream of helium. Typically, 2–15% RCY was obtained (not decay corrected) in a synthesis time of 1 h, including drying of fluorine-18.

### 3.7. Labeling of [^18^F]FVIIai

Active site inhibited factor VII (1.2 mg in 500-μL HEPES buffer, pH 7.8) was added to purified and dried [^18^F]SFB and stirred gently for 30 min at room temperature. The final purification of [^18^F]FVIIai was conducted on a PD-10 desalting column eluting with phosphate buffered saline collecting fractions of 0.5 mL. Fractions 4 to 8 contained [^18^F]FVIIai corresponding to 11% incorporation of [^18^F]SFB.

## 4. Conclusions

A one-step labeling procedure for [^18^F]SFB was developed. RCYs with a maximum RCY of 15% could be reached in less than 1 h. The product was radiochemical pure. No release of radioactive side-product was observed. Unfortunately, large variations in the yield were observed. This is due to hydrolysis of the active ester into [^18^F]FBA ([^18^F]3). Future efforts will be directed to minimize the hydrolysis step and increase the reliability of the reaction.

## Figures and Tables

**Table 1:** One-step labeling procedure to synthesize [^18^F]SFB.

Precursor	RCC [%]		RCY [%]	
**4a**	1–33	n = 26	2–15	n = 7
**4b**	5	n = 3	-	-
**4c**	8–35	n = 2	1–2	n = 2
**4d**	6	n = 2	-	-

RCC = radiochemical conversion; RCY = radiochemical yield.

**Table 2:** Comparison of published [^18^F]SFB syntheses.

Method	RCY (d.c.) [%]	RCY (n.d.c.) [%]	RCP [%]	Synthesis Time [min]
This work	3–22	2–15 (average 4)	>95	<60
Vaidyanathan [3]	30–35	18–21	n.d.	80
Thonon et al. [4]	42 ± 3	29 ± 2	98	57
Li et al. [5]	80 ± 3	60 ± 2	97	45
Ackermann et al. [7]	75–85	51–59	95–98	58

n.d. = not determined; RCY = radiochemical yield; RCP = radiochemical purity; d.c. = decay corrected; n.d.c. = non-decay-corrected

## Supplementary Materials

Analytical HPLC and NMR data, additional experimental data including analytical HPLC conditions and reaction optimization attempts are reported.
