# Supplementary material for: One-Step Synthesis of N-Succinimidyl-4-[18F]Fluorobenzoate ([18F]SFB)
Source: Molecules. 2019 Sep 22;24(19):3436. doi: 10.3390/molecules24193436 (PMC6804101; doi:10.3390/molecules24193436)

One step synthesis of N-succidimidyl-4-[^{18}F]-fluorobenzoate ([^{18}F]SFB)

Ida Nymann Petersen[a,b,c], Jacob Madsen[b], Christian Bernard Matthijs Poulie[c], Andreas Kjær[b,c] and Matthias Manfred Herth[a,b]

[a]*Department of Drug Design and Pharmacology, Faculty of Health and Medical Sciences, University of Copenhagen, Jagtvej 160, DK-2100 Copenhagen, Denmark*

[b]*Department of Clinical Physiology, Nuclear Medicine & PET, Rigshospitalet, Blegdamsvej 9, DK-2100 Copenhagen, Denmark*

[c]*Cluster for Molecular Imaging, Department of Biomedical Sciences, University of Copenhagen, Blegdamsvej 9, DK-2100 CopenhagenDenmark*

SI Table 1: Optimization of time (used conditions for the experiment: K_2CO_3 (1.2 mg) / K_{222} (10 mg)/DMF (0.3 ml), 130ºC)

	RCC [%]		
	5 min	10 min	20 min
[^{18}F]SFB	9	6	4
[^{18}F]3 ([^{18}F]FBA)	3	12	11

SI Table 2: Experimental conditions used to synthesize [^{18}F]SFB from 4a (5 mg)

Entry	Base/cryptand	Solvent 0.3 mL	Additives	Temperature	Time [min]	RCC [%]**	Mean ± SD	n
1	1.2 mg K_2CO_3/10 mg K_{222}	DMF	-	130ºC	5	2-33	17.8±17	26
2	1.1 mg $KHCO_3$/ 10 mg K_{222}	ACN	t-BuOH (100 µL)	130ºC	5	0	-	1
3	1.1 mg $KHCO_3$/10 mg K_{222}	DMF	-	130ºC	5	0	-	1
4	1.1 mg $KHCO_3$/ 10 mg K_{222}	DMF	Pyridine (100µL)	130ºC	5	0	-	1
5	KOTf 10mg /1.2 mg K_2CO_3	DMF	-	130ºC	5	0	-	1
6	TEAB 10 mg	DMF	-	130ºC	5	0	0	2
7	Cs_2CO_3 5 mg	DMF	-	130ºC	5	0	-	1
8	1.2 mg K_2CO_3/ 10 mg K_{222}	ACN	-	90ºC	5	0	-	1
9	1.2 mg K_2CO_3/10 mg K_{222}	DMSO	-	130ºC	5	0	-	1
10	1.1 mg $KHCO_3$/ 10 mg K_{222}	DMF	TEMPO 1 mg	130ºC	5	0	-	1
11	K_2CO_3/K_{222}	DMF	TEMPO, 1mg	130ºC	5	3-17	12±14	11
12	K_2CO_3/18-crown-6	DMF		130ºC	5	4	-	1
13	1.2 mg K_2CO_3/10 mg K_{222}	DMF	-	90ºC*	10	0	-	1
14	5 mg K_2CO_3/10 mg K_{222}	DMF		130ºC	5	0	-	1
15	1.2 mg K_2CO_3/ 10 mg K_{222}	DMF	-	90ºC	5	0	-	1
15	1.2 mg K_2CO_3/ 10 mg K_{222}	DMF	-	140ºC	5	0	-	1

*Microwave heating; ** trace amount of acid sometimes detected if yield was 0.

Example of a semip-preparative chromatogram of [^{18}F]SFB

a) Radiochromatogram

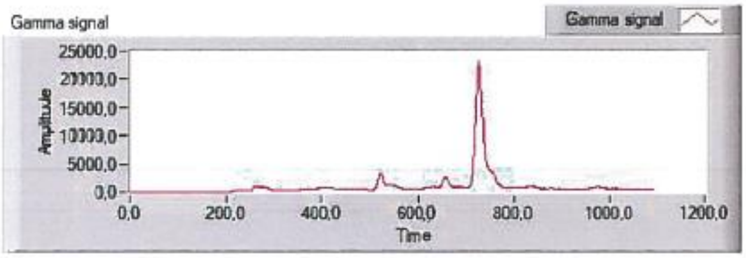

b) UV-chromatogram

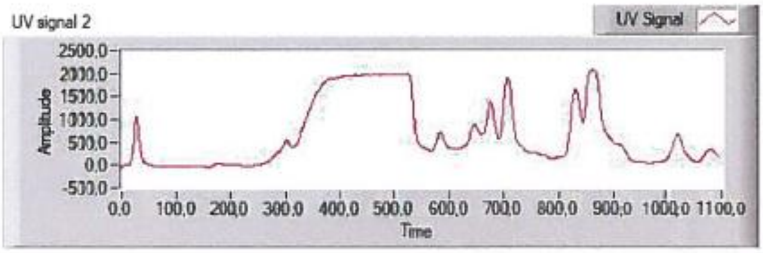

Analytical HPLC chromatograms

Method 1: *C18 LUNA (phenomenex) column, 5 µm, 250 × 4.6 mm in 2-mL/min solvent flow. A gradient system with two eluents, A and B, was used, with the fraction of B varying from 0% to 100% over 15 min. A =* H_2O*, 0.1% TFA; B = MeCN :* H_2O*, 0.1% TFA*

Method 2: *C4 Grace Vydac column, 5 µm, 250 × 4.6 mm in 1-mL/min solvent flow. A gradient system with two eluents, A and B, was used, with the fraction of B varying from 0% to 100% over 25 min. A =* H_2O*, 0.1% TFA; B = MeCN :* H_2O*, 0.1% TFA*

Reference of SFB, Method 1

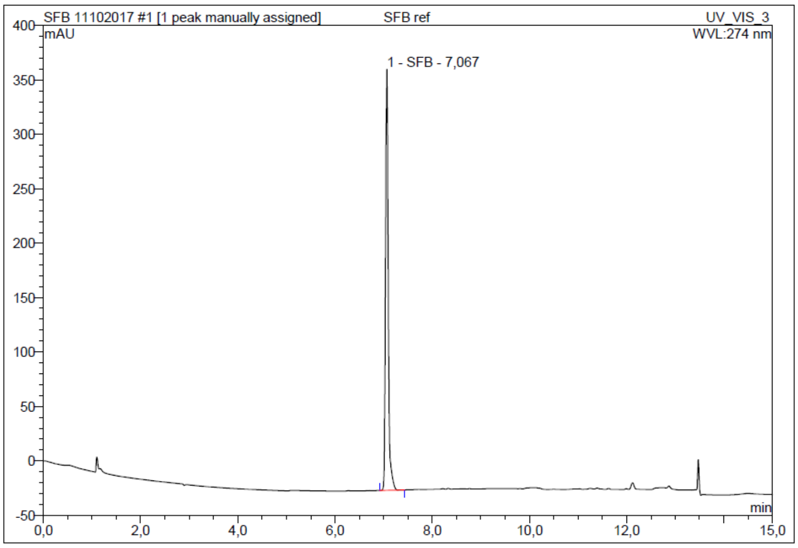

No.	Ret.Time min	Peak Name	Height mAU	Area mAU*min	Rel.Area %	Amount	Type
1	7,07	SFB	386,994	25,090	100,00	n.a.	BMB^
Total:			386,994	25,090	100,00	0,000	

SFB crude reaction mixture, Method 1

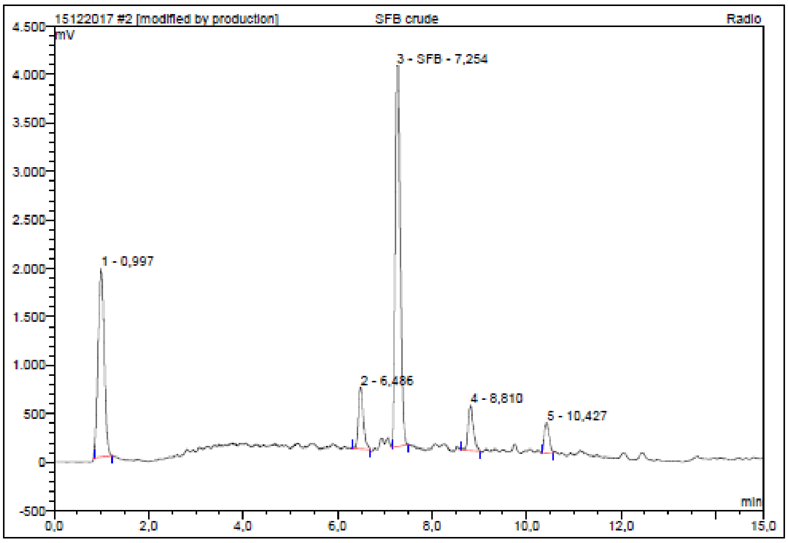

No.	Ret.Time min	Peak Name	Height mV	Area mV*min	Rel.Area %	Amount	Type
1	1,00		1949,240	299,292	31,46	5,000	BMB
2	6,49	n.a.	634,480	76,812	8,07	n.a.	BMB
3	7,25	SFB	3931,943	478,211	50,27	4,205	BMB
4	8,81	n.a.	467,396	60,362	6,35	n.a.	BMB*
5	10,43	n.a.	309,864	36,579	3,85	n.a.	BMB
Total:			7292,922	951,256	100,00	9,205	

SFB purified, Method 1

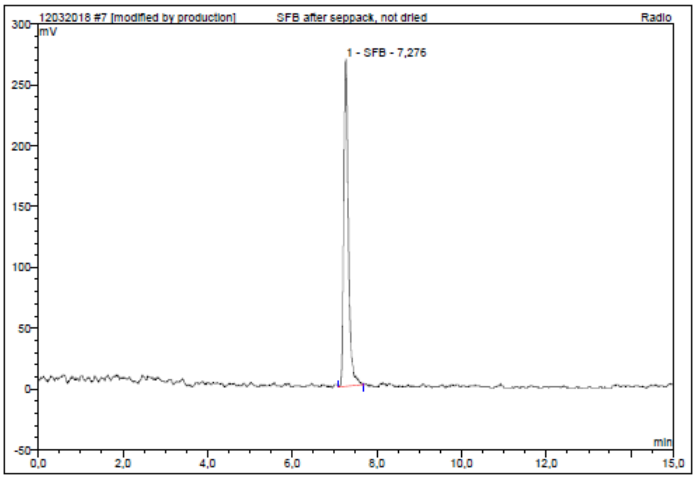

No.	Ret.Time min	Peak Name	Height mV	Area mV*min	Rel.Area %	Amount	Type
1	7,28	SFB	268,610	34,185	100,00	3,623	BMB*
Total:			268,610	34,185	100,00	3,623	

SFB purified and spiked with cold reference, Method 1

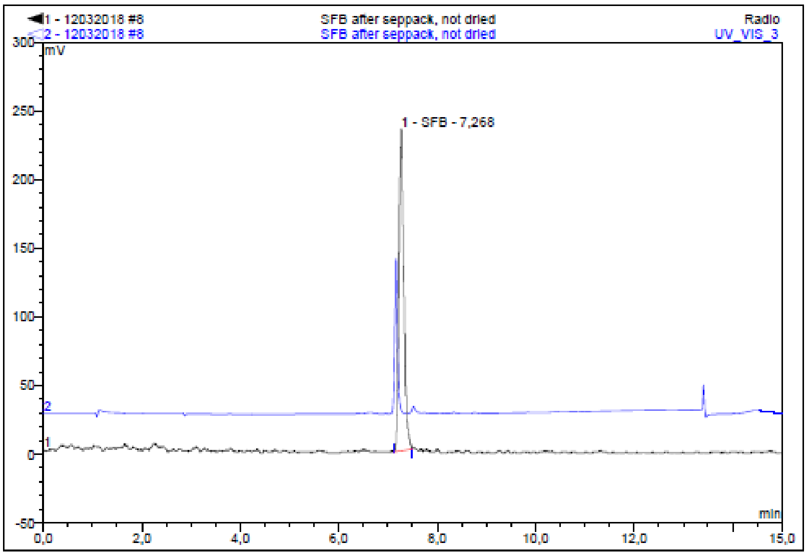

No.	Ret.Time min	Peak Name	Height mV	Area mV*min	Rel.Area %	Amount	Type
1	7,27	SFB	234,364	27,962	100,00	2,963	BMB
Total:			234,364	27,962	100,00	2,963	

SFB reacted with [^{18}F]FVIIai, Method 2

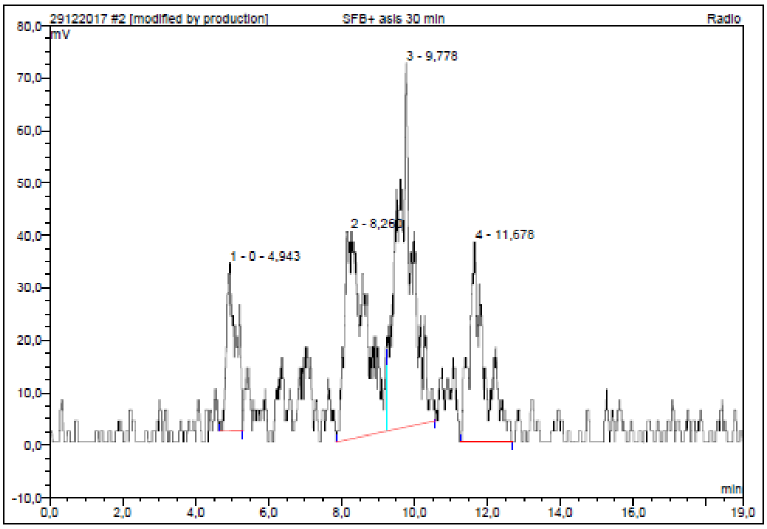

No.	Ret.Time min	Peak Name	Height mV	Area mV*min	Rel.Area %	Amount	Type
1	4,94	0	32,072	9,641	10,81	n.a.	BMB*
2	8,26	n.a.	39,563	27,299	30,60	n.a.	BM *
3	9,78	n.a.	69,351	33,697	37,78	n.a.	MB*
4	11,68	n.a.	38,202	18,564	20,81	n.a.	BMB*
Total:			179,188	89,201	100,00	0,000	

SFB reacted with [^{18}F]FVIIai and purified by PD10, Method 2

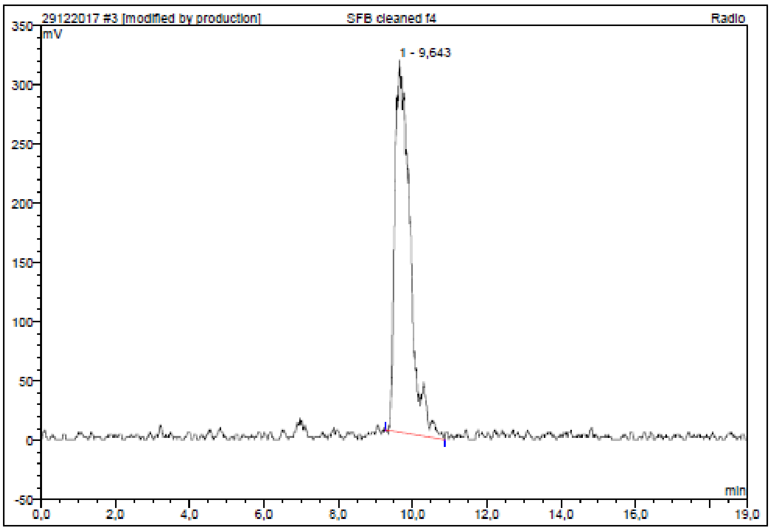

No.	Ret.Time min	Peak Name	Height mV	Area mV*min	Rel.Area %	Amount	Type
1	9,64	n.a.	314,134	149,469	100,00	n.a.	BMB*
Total:			314,134	149,469	100,00	0,000	

SFB reacted with ASIS and purified by PD10 overlay, Method 2

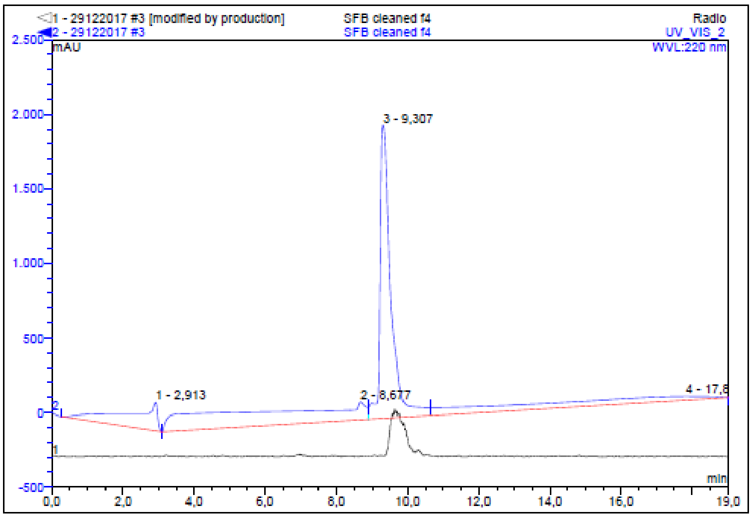

No.	Ret.Time min	Peak Name	Height mAU	Area mAU*min	Rel.Area %	Amount	Type
1	2,91	n.a.	188,727	188,493	11,10	n.a.	BMB
2	8,68	n.a.	117,921	527,790	31,08	n.a.	BM
3	9,31	n.a.	1968,392	702,500	41,36	n.a.	M
4	17,83	n.a.	25,293	279,539	16,46	n.a.	MB
Total:			2300,333	1698,323	100,00	0,000	

NMR spectra

^{1}H-NMR of SFB-precursor (4a)

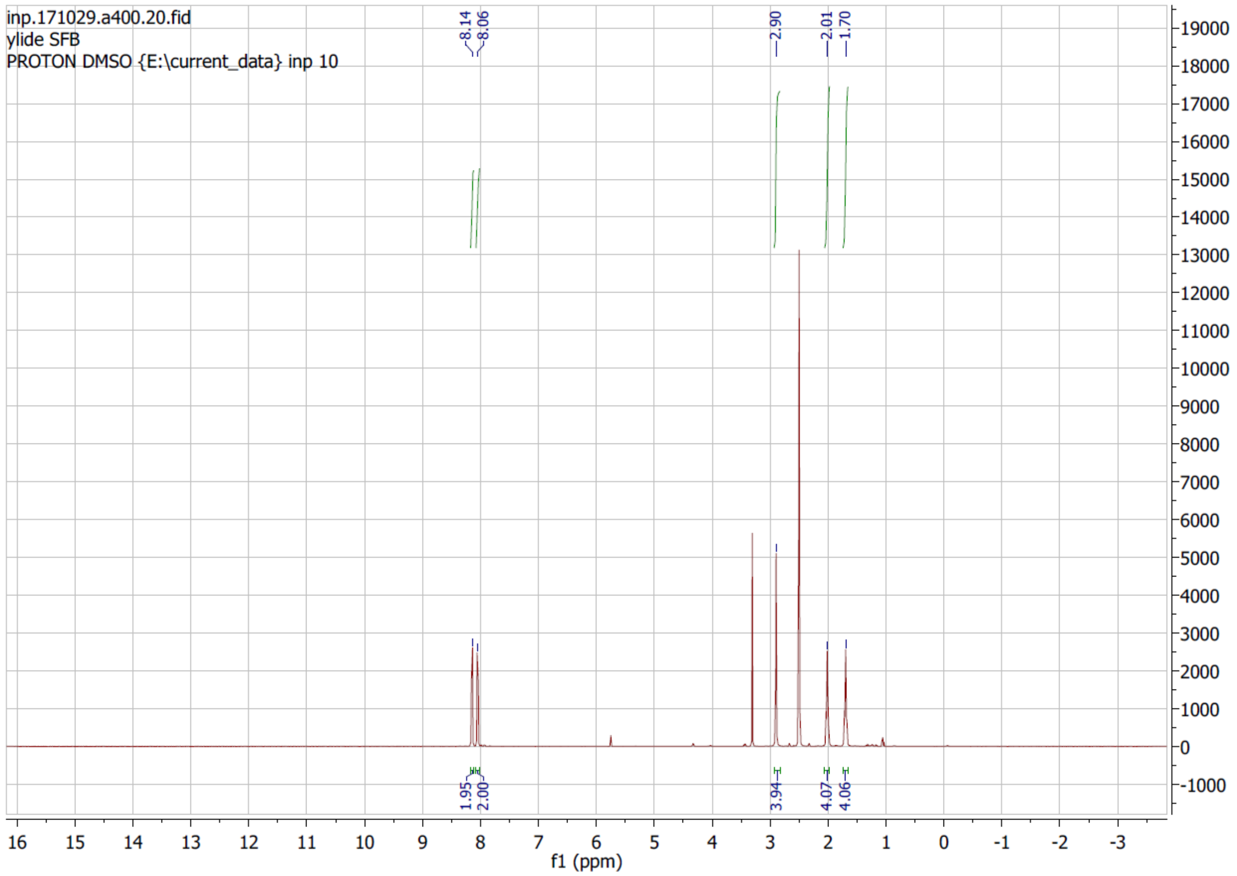

^{13}C-NMR of SFB-precursor (4a)

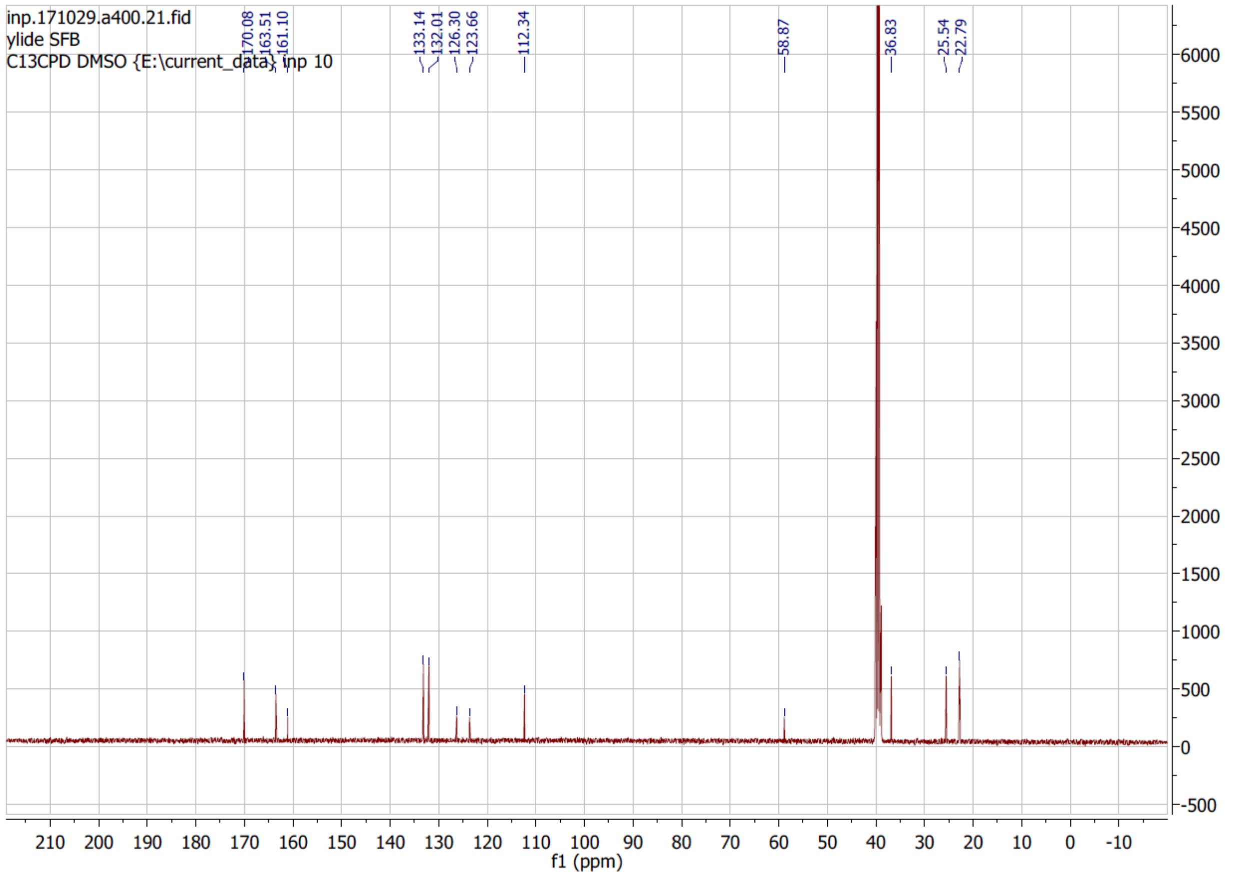

^{1}H-NMR of SFB-precursor (4b)

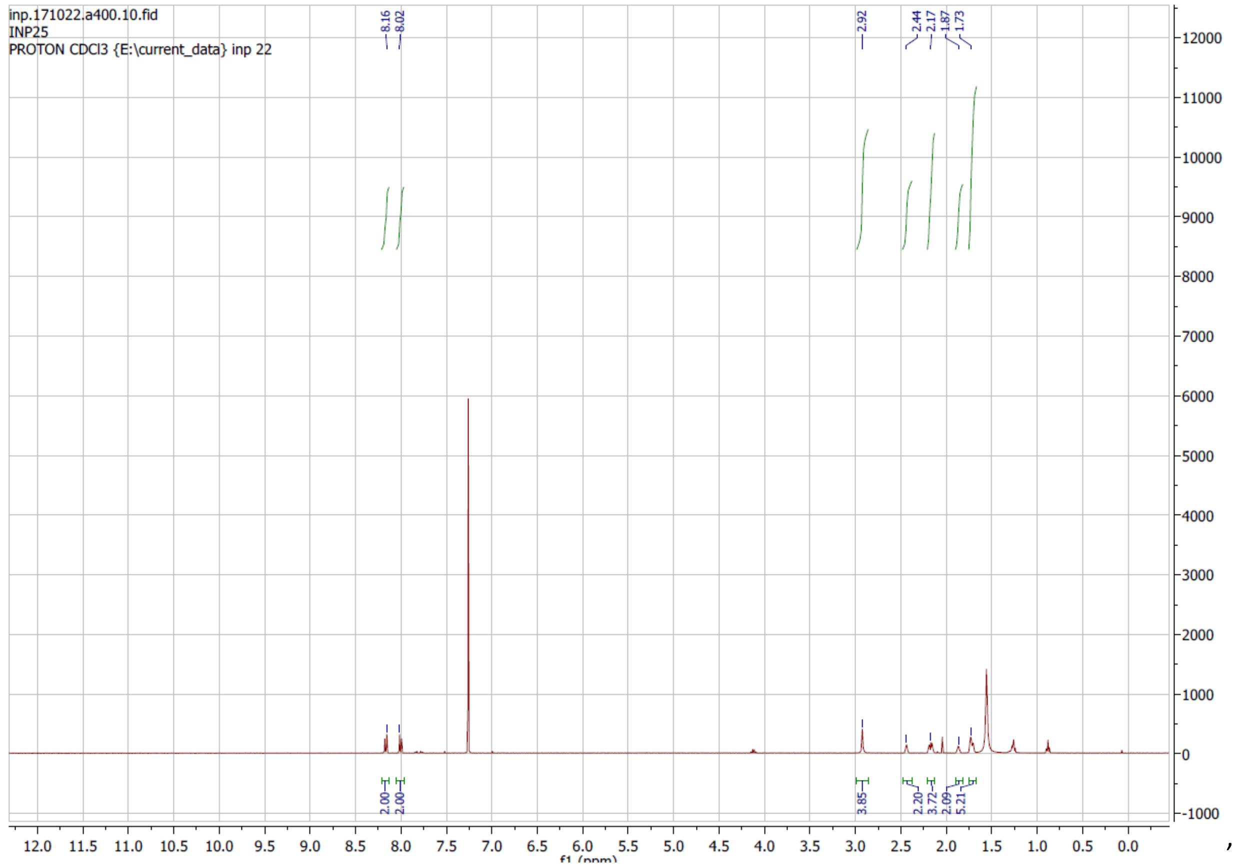

^{13}C-NMR of SFB-precursor (4b)

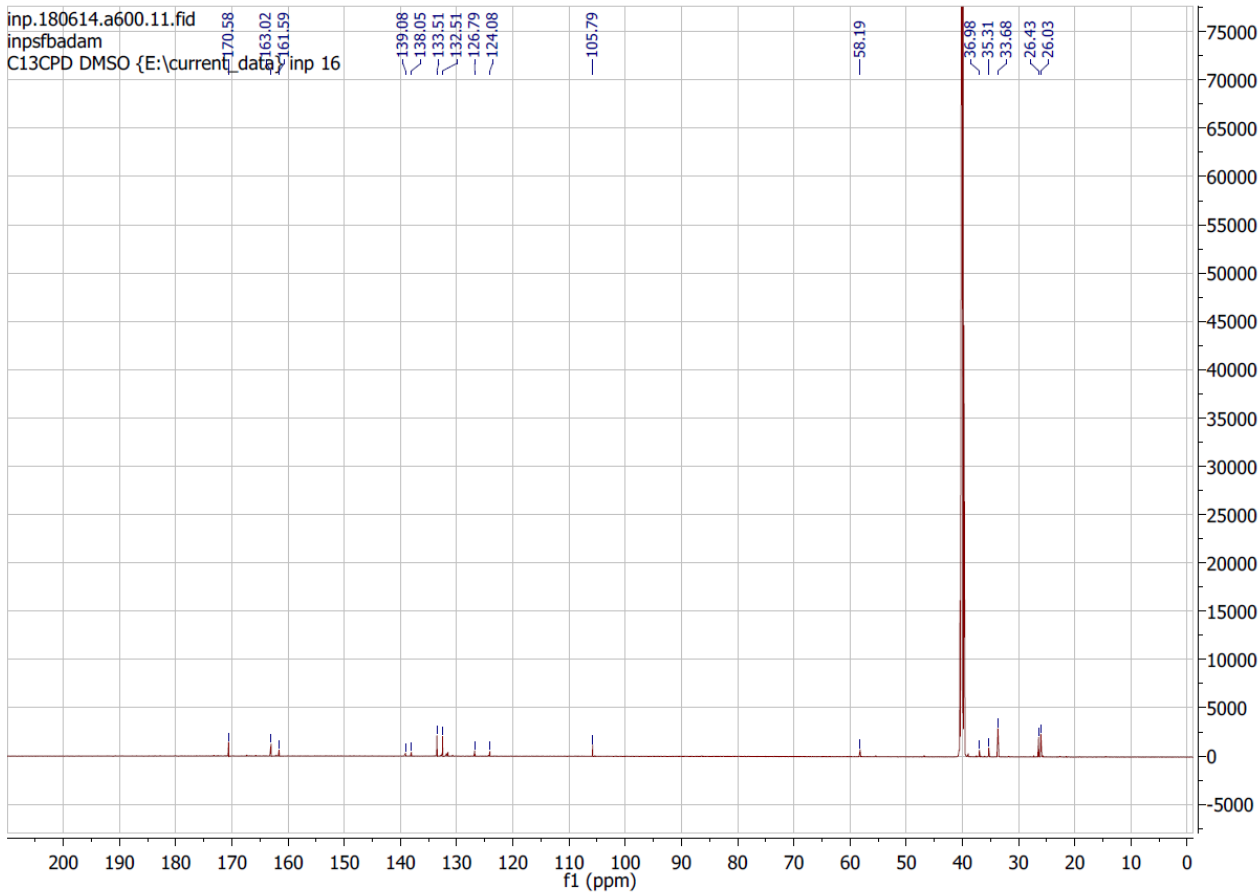

^{1}H-NMR of SFB-precursor (4c)

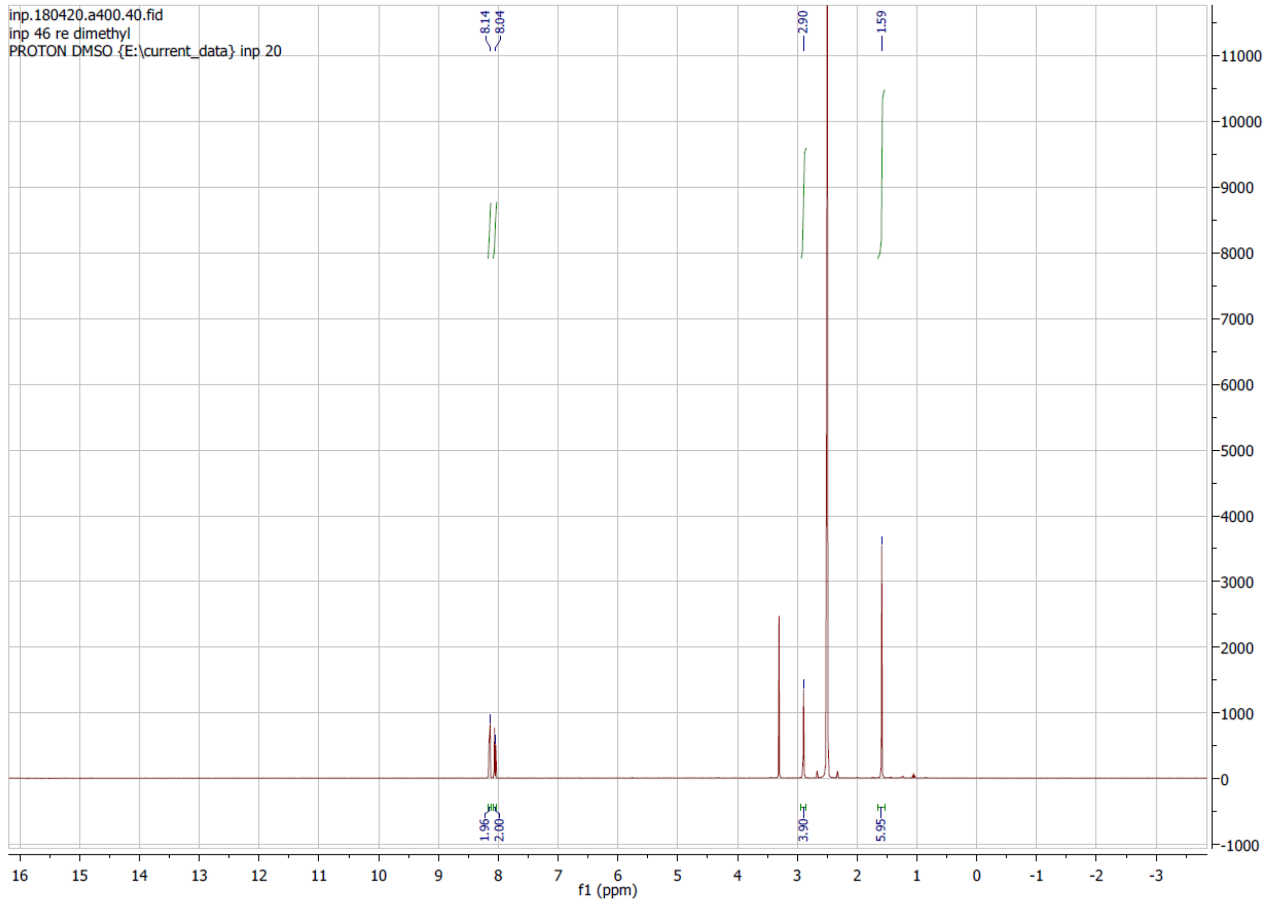

^{13}C-NMR of SFB-precursor (4c)

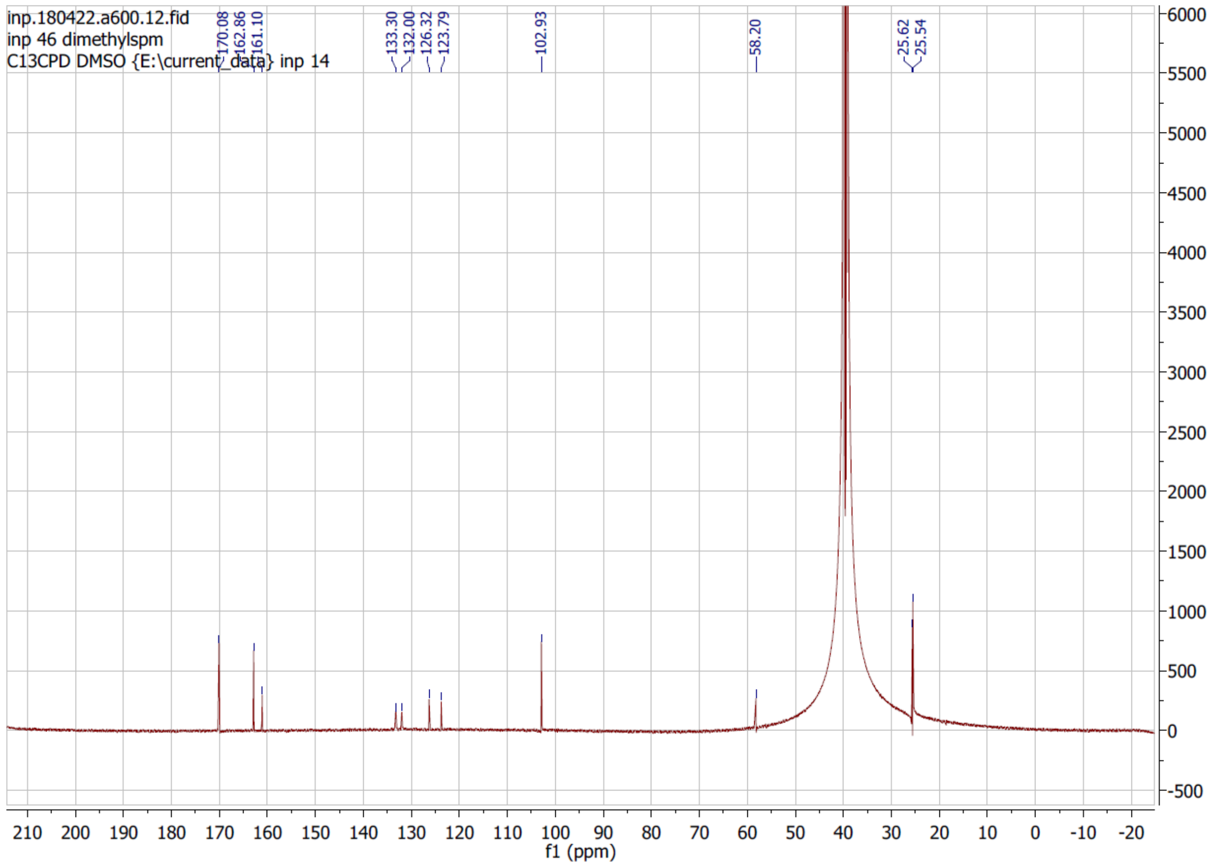

^{1}H-NMR of SFB-precursor (4d)

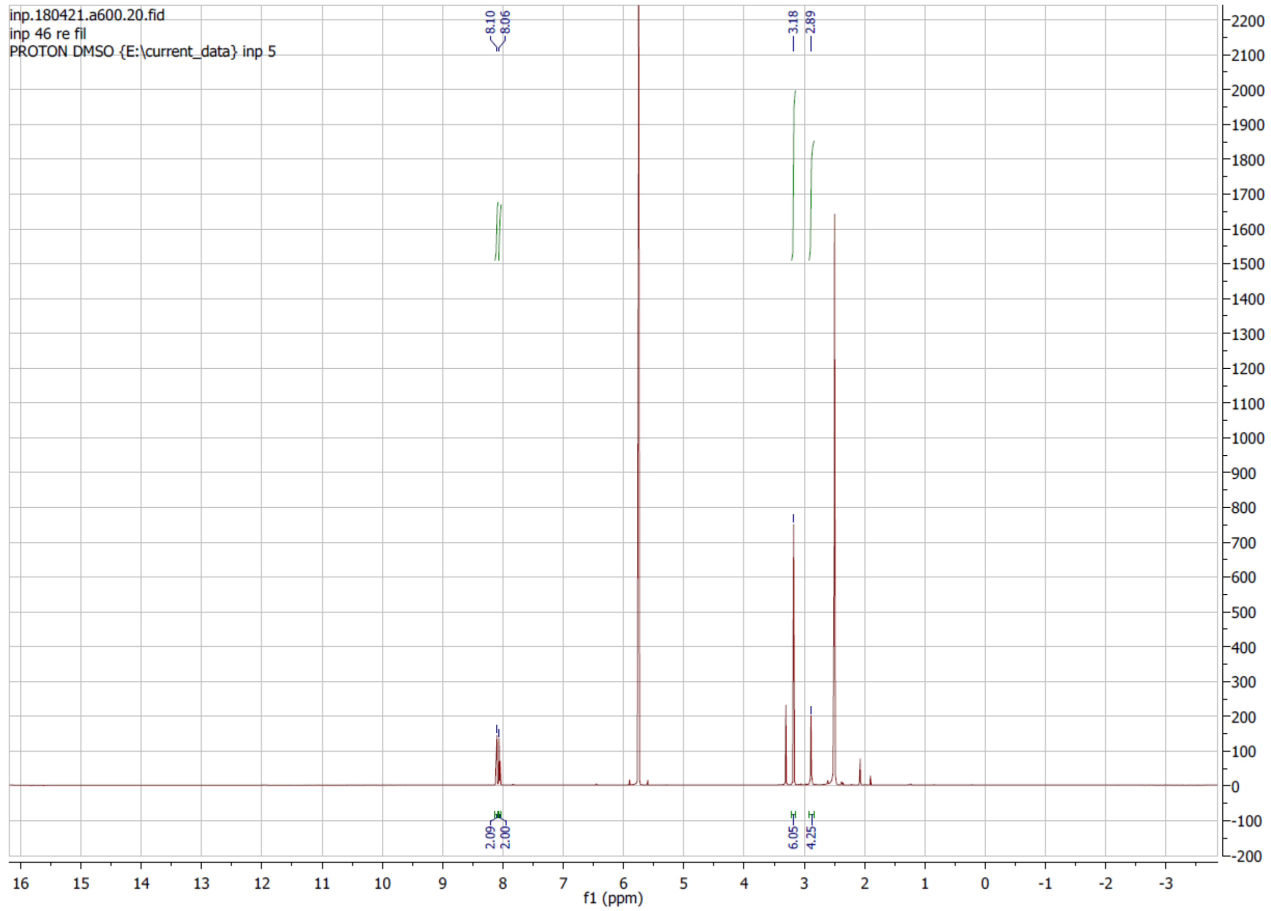

^{13}C-NMR of SFB-precursor (4d)

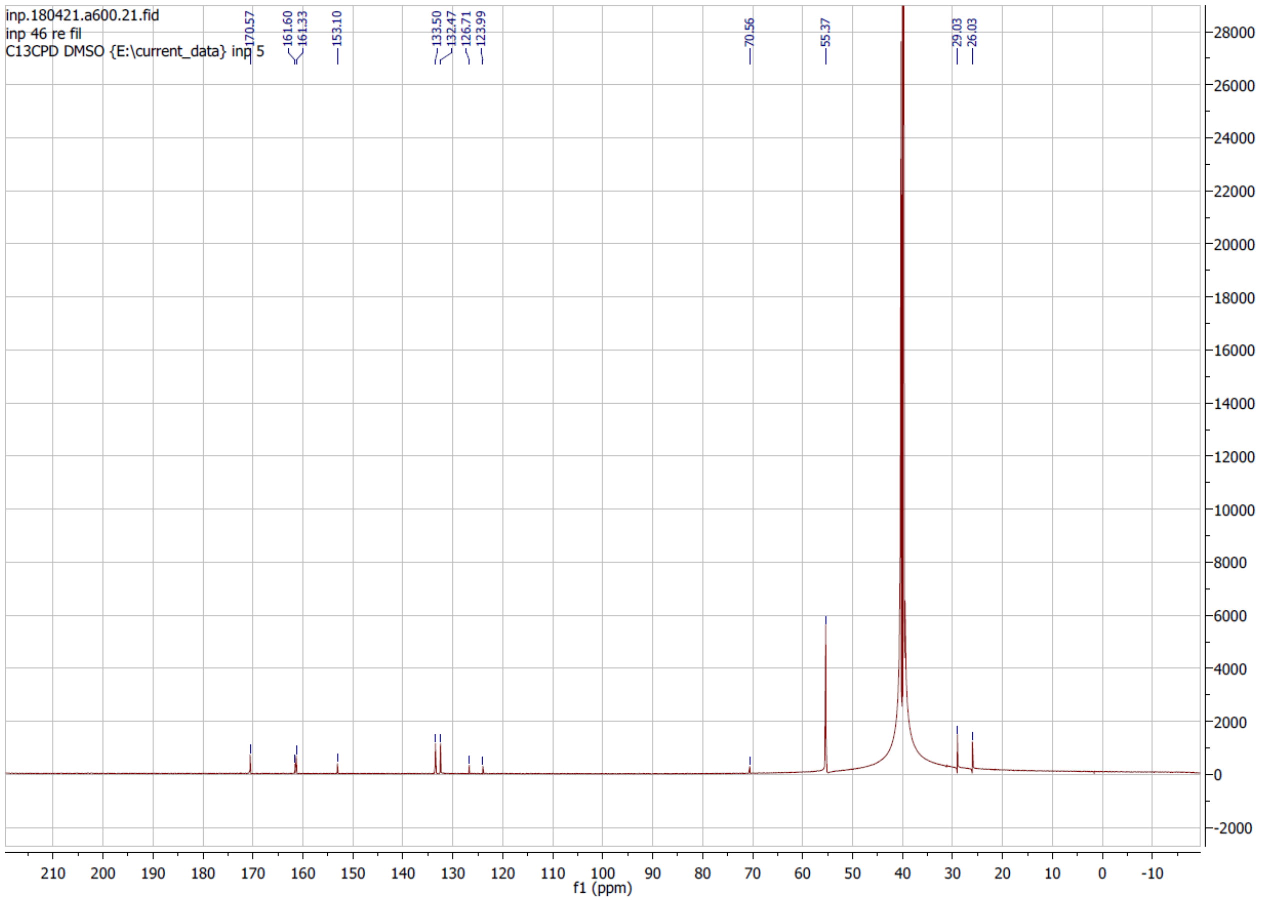

Supplement: Supplementary file 1 [file molecules-24-03436-s001.pdf]
